# Implementation and Delivery of a Comprehensive Bereavement Program in Pediatric Palliative Oncology

**DOI:** 10.3390/children13091163

**Published:** 2026-08-29

**Authors:** Yash C. Jani, Anna Lange, Miracle Naylor, Katherine Lee, Linda M. Radbill, Katharine E. Brock

**Affiliations:** 1 Department of Medicine, Medical College of Georgia, Augusta, GA 30912, USA; yjani@augusta.edu; 2 Aflac Cancer and Blood Disorders Center, Children’s Healthcare of Atlanta, Atlanta, GA 30329, USAkatherine.lee2@choa.org (K.L.);; 3 Department of Pediatrics, Emory University School of Medicine, Atlanta, GA 30322, USA

**Keywords:** pediatric oncology, bereavement, grief, pediatric palliative care, quality improvement, end-of-life care, bereaved families, psychosocial oncology

## Abstract

**Highlights:**

**What are the main findings?**

- A structured, iteratively developed bereavement program achieved annual delivery rates of 93–100% for individual components across 403 bereaved families over nine years.
- Comprehensive bereavement care did not require a fully formed program at the outset; it evolved incrementally from a single tracked activity into a six-component program.

**What are the implications of the main findings?**

- Programs at any stage of development can start small, track their progress, and add components as infrastructure and staffing allow.
- Family-reported outcomes are needed to determine which bereavement components are received, valued, and meaningful to families.

**Abstract:**

**Background/Objectives**: Bereavement care is widely endorsed as a standard of pediatric oncology and palliative care, yet it remains inconsistently implemented across pediatric oncology programs in the United States, and few programs have reported longitudinal delivery metrics. We aim to describe the iterative development and delivery of a multi-component bereavement program within an outpatient pediatric palliative oncology (PPO) program, characterize the population served, and benchmark delivery against national guidelines. **Methods**: This was a retrospective descriptive analysis of bereavement component delivery for PPO program patients who died between 2017 and 2026, using an iterative quality improvement framework. Program components (calls, condolence cards, packets, books) were tracked prospectively in a registry. Annual completion rates and timeliness were calculated for each family-facing component, and the cohort was characterized by demographic, clinical, and end-of-life variables. Data were reviewed for completeness at extraction; missing values were rare and are noted in the relevant tables. **Results**: A cohort of 403 deceased patients was included (41% Black, 15% Hispanic/Latino, 50% Medicaid-insured). The program evolved from informal bereavement phone calls (completion 74–81%) during 2017–2019 to a standardized, six-component program by 2020, reaching 93–100% annual delivery for most eligible components during 2022–2025. Early outreach occurred promptly (median 6 days after death for phone calls and condolence cards), and longer-term components were delivered within their intended windows. **Conclusions**: Comprehensive bereavement was developed over time without a fully formed infrastructure at initiation. This structured program began with one feasible, trackable activity and added components incrementally, achieving highly sustainable delivery that ultimately informed broader institutional practice.

## 1. Introduction

Cancer remains a leading cause of disease-related death among children and adolescents in the United States. Although outcomes have improved substantially over the past five decades, an estimated 1820 children and adolescents will die from cancer in 2026, leaving many families to navigate the enduring consequences of pediatric cancer bereavement [1]. The death of a child is among the most devastating forms of loss and increases the risk of prolonged grief disorder, depression, anxiety, posttraumatic stress disorder (PTSD), and impaired psychosocial functioning in parents and siblings [2,3,4,5]. Among bereaved parents of children with cancer, clinically significant anxiety, depression, and PTSD are common; 10–25% of parents bereaved by cancer ultimately experience debilitating grief symptoms that may persist or intensify beyond the first year after death [2,5]. Siblings are also profoundly affected, with a recent meta-analysis demonstrating high rates of prolonged grief-related symptoms among young people who experienced the death of a parent or sibling from cancer [4].

Although ongoing clinician contact after death is consistently identified as meaningful and healing, bereaved families frequently describe feeling abandoned by the medical team after their child’s death [6,7,8]. For some parents, the abrupt discontinuation of contact with clinicians who cared for their child represents a “second loss,” compounding grief and isolation [7,8]. Phone calls, condolence letters, family meetings, and other forms of remembrance may help families feel that their child is remembered, provide continuity with the care-team, and support meaning-making during early bereavement [9,10]. Clinicians can provide brief moments of education that validate caregivers’ experiences of grief while offering practical assistance in seeking support. These findings highlight the important role healthcare institutions can play in supporting families beyond a child’s death.

Multiple professional organizations endorse bereavement care as a standard of pediatric oncology and palliative care. The American Academy of Pediatrics (AAP) recommends structured family support, including phone calls, condolence letters, and memorial services [11,12,13]. The National Comprehensive Cancer Network (NCCN) Palliative Care Guidelines recommend formal condolence, referral to bereavement services, and staff debriefing [14]. NCCN Adolescent and Young Adult Oncology Guidelines specify that programs should provide bereavement follow-up with at least one contact after death [14]. The Psychosocial Standards for Children with Cancer and Their Families identify bereavement care as one of 15 core psychosocial standards and emphasize that bereaved families should receive continued support and access to appropriate bereavement resources after a child’s death. More recently, the Society of Critical Care Medicine guidelines recommended bereavement care processes for children and families that begin during end-of-life care and continue after death, with emphasis on reducing disparities in end-of-life care [15].

Despite broad endorsement, bereavement care remains inconsistently implemented across pediatric oncology programs in the United States. In a national survey of Children’s Oncology Group (COG) centers in the United States, only 69% of programs sent a condolence card or letter and only 50% made a bereavement phone call to assess psychosocial status after a child’s death; in-person meetings, psychotherapy, and support groups were offered even less frequently [16]. Nearly a decade after publication of the Psychosocial Standards, the iSTEPPP provider-report study found that supportive care and bereavement remained among the lowest-rated domains for provider-reported delivery of standards-consistent psychosocial care across pediatric oncology programs, with limited improvement over time [17]. These findings suggest that bereavement care, although widely recognized as important, remains difficult to operationalize and sustain as a consistent component of pediatric oncology care. This implementation gap likely reflects multiple barriers, including limited reimbursement, lack of dedicated personnel, competing clinical demands, absence of standardized workflows, and limited evidence on which bereavement practices are most meaningful to families.

Several institutions have described components of their pediatric bereavement programs, but few have reported longitudinal delivery metrics. The St. Jude Children’s Research Hospital bereavement program, developed in partnership with bereaved parents, provides a comprehensive model of clinical support, parent-created materials, and opportunities for family engagement in research and education, but longitudinal delivery-rates are not yet reported [18,19]. Other programs highlight the importance of systematic, trackable outreach [20] that is individualized to family needs [21,22] and includes written materials, professional support, and connection with other bereaved parents. Collectively, these studies demonstrate the value of bereavement programming while highlighting the need for implementation models that are structured, longitudinal, measurable, and responsive to family needs.

To address this gap, we describe the development and delivery of a multi-component bereavement program within a large outpatient pediatric palliative oncology (PPO) program. The aims of this quality improvement project were to: (1) describe the iterative development of the program from 2017 to 2026; (2) report delivery metrics, including reach and timeliness, for each bereavement component; (3) characterize the population served; and (4) contextualize the program within national guidelines and published benchmarks.

## 2. Materials and Methods

### 2.1. Setting and Population

This study was conducted at the Aflac Cancer and Blood Disorders Center at Children’s Healthcare of Atlanta (CHOA), a large academic pediatric oncology center caring for about 480 patients with newly diagnosed cancer annually. The outpatient pediatric palliative oncology (PPO) program started in 2017 and provides longitudinal palliative care, including symptom management, goals-of-care communication, advanced care planning, and care coordination. The outpatient PPO program operates alongside a separate inpatient pediatric palliative care (PPC) consult service, which, as of 2022, includes an embedded bereavement coordinator.

The study population included all pediatric patients followed by the outpatient PPO program who died between 2017 and 2026. No exclusion criteria were applied; all patients with a documented death during the study period were included in the analysis.

### 2.2. Program Description: Iterative Development

**Figure 1:**
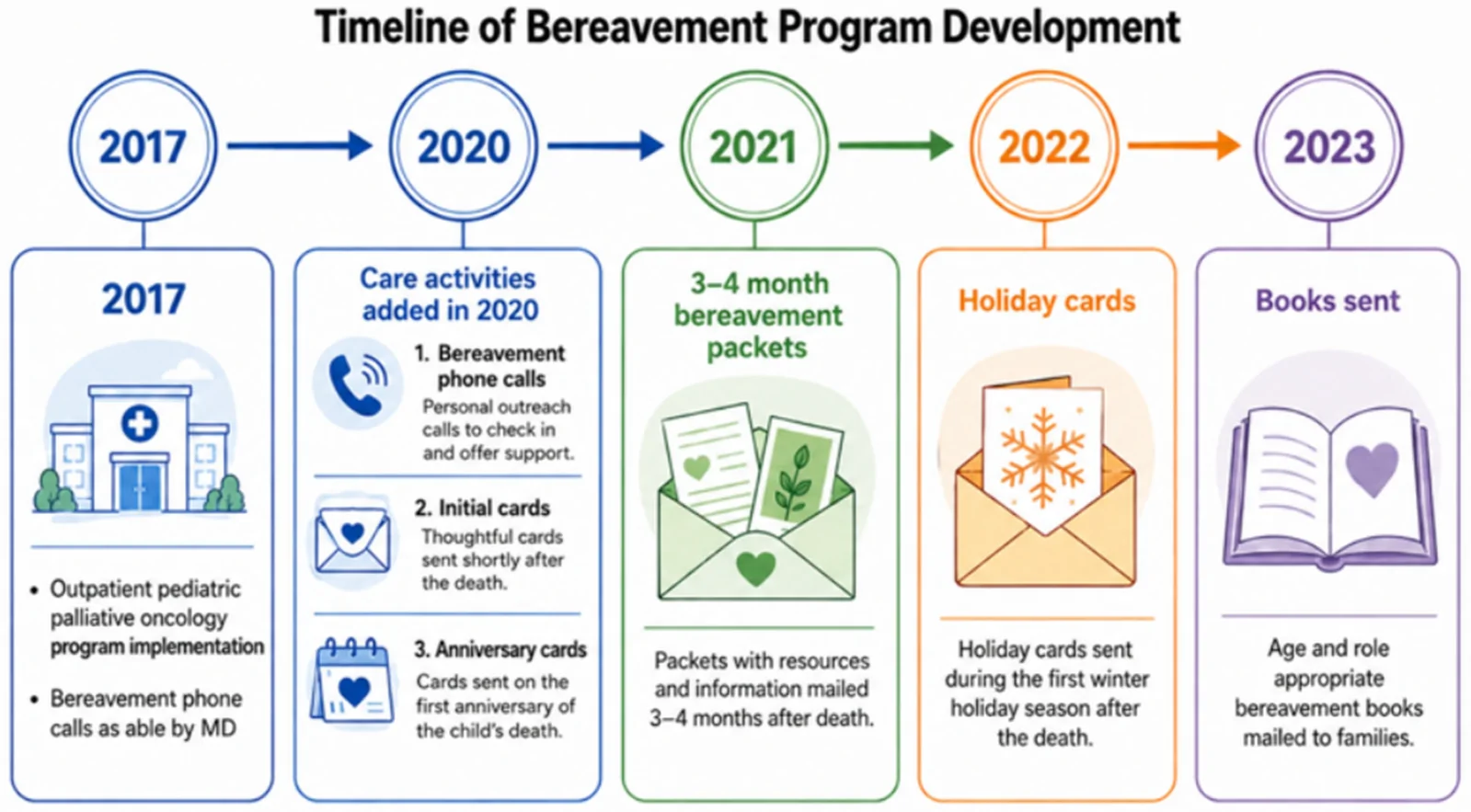
Timeline of patient-facing bereavement program development to date. Sequential development of family-facing and staff-facing bereavement support activities within the outpatient pediatric palliative oncology program from 2017 to 2026.

The bereavement program described in this quality improvement project was developed and implemented within the outpatient PPO program, evolving in two phases: an informal pre-implementation period from 2017 to 2019 and a structured, multi-component program implemented sequentially from 2020 to 2026 (Figure 1).

Prior to a child’s death, the PPO team provided informal anticipatory grief and bereavement support tailored to each family’s needs, including advance care planning conversations, anticipatory grief counseling, and sibling support. Materials utilized included advance care planning resources (e.g., Voicing My Choices), educational resources (e.g., When the Time Comes), and age-appropriate books (e.g., The Invisible String). This pre-death support was not standardized and is not reflected in the post-death delivery metrics reported here.

During the pre-implementation baseline period, two components were informally developed: bereavement phone calls conducted by the PPO physician were the only systematically tracked activity, and outreach was not yet standardized. The PPO team also helped plan the annual Celebration of Life event beginning in 2018. This in-person event (held virtually during 2020–2021 due to COVID-19) invites families—parents, siblings, and extended relatives—whose child died in the preceding calendar year. The event includes welcoming remarks, poetry, music, a reading of the names of children who died, opportunities for families to share memories, a structured activity, and fellowship with other bereaved families. Siblings participate in a separate age-appropriate activity facilitated by a child life specialist.

Beginning in 2020, the PPO team implemented a structured program, adding components over time based on feasibility and family needs. Bereavement phone calls were standardized to a goal of contact within two weeks of death (at least two attempts, with a voicemail if unreachable) and documented in the electronic health record (EHR). During 2017–2019, bereavement calls were conducted by the PPO physician; beginning in 2020, calls were conducted by either the PPO physician or the advanced practice provider (APP). Initial handwritten condolence cards were introduced and mailed within one week of death; all members of the PPO team who knew the child wrote a note and signed the card prior to mailing. Handwritten one-year anniversary cards were filed concurrently to support reliable delivery at the first anniversary of the child’s death.

In 2021, the team added a 3–4-month-bereavement resource packet mailed directly to the family’s home (Supplement Table S1). The packet included (1) a typed personalized condolence letter signed by the team, (2) a community resource list, (3) age-specific sibling grief information, (4) a *When You Are Grieving* booklet, and (5) the Mourner’s Bill of Rights. In 2022, annual holiday cards were introduced for every family whose child died during the previous 12 months, signed by the PPO and oncology teams. Starting in 2023, age-appropriate bereavement books for parents and siblings were mailed based on sibling ages. Starting in 2026, bereaved parent support groups are being offered. Groups are facilitated by PhD-level pediatric palliative care psychologists and master’s-level therapists. Parents and caregivers whose child died at least six months prior are eligible and are invited via a mailed flyer with a QR code directing them to complete an interest form; a mental health clinician then conducts a screening phone call prior to group acceptance. Each cohort consists of approximately six sessions over approximately two months, grounded in evidence-based principles of cognitive-behavioral therapy. The first group is being scheduled at the time of writing.

In parallel, the program incorporated practices to support staff and clinician awareness. By 2020, the team routinely added a death flag to the EHR chart, notably for deaths at home, and notified relevant clinical teams by email. Starting in 2021, the PPO team held an annual remembrance night, and in 2023 established a monthly neuro-oncology bereavement pause, led by the PPO APP at the monthly neuro-oncology administrative meeting. The full multidisciplinary team—including oncologists, palliative care physicians, pathologists, APPs, social workers, schedulers, school teachers, case managers, research coordinators, and administrative staff—is invited. Each child’s name is read aloud, followed by open discussion in which team members share memories and reflections. In 2024, the team started tracking all deaths and tasks in Microsoft Teams using a shared task list which includes: the team member making the bereavement phone call, whether the call is completed and documented in the EHR, whether initial condolence cards are signed/sent, whether the patient’s REDCap registry has been updated and closed, whether the patient has been marked as deceased in the EHR, and whether emails have been sent to relevant clinical teams (Supplement Figure S1). Additionally, based on parent feedback about the difficulty of losing access to MyChart 11.8.4 at the time of death, the PPO team worked with Information Technology/EPIC to allow six months of MyChart access following the death.

### 2.3. Study Design

This quality improvement project was a retrospective descriptive analysis of bereavement outreach delivery within an iterative quality improvement (QI) framework. Reporting was guided by the Standards for Quality-Improvement Reporting Excellence (SQUIRE 2.0) guidelines [23]. The program used the Institute for Healthcare Improvement Model for Improvement as its guiding framework, applying sequential Plan-Do-Study-Act (PDSA) cycles to add, test, and refine bereavement components based on team assessment, operational feasibility, and delivery data [24]. Key drivers guiding program development included: (1) providing intermittent, growth-oriented, and meaningful remembrance for families; (2) bridging surviving family members to community resources; and (3) enabling proactive tracking of bereavement components. Decisions about implementation sequence were informed by the bereaved parent literature, perceived clinical impact, and funding feasibility [24]. Bereavement outreach has been tracked prospectively in real time in a secure REDCap database since 2017 (Supplement Figure S2); the present analysis is a retrospective review of these prospectively collected data, with the dataset extracted on 22 May 2026, for this study [25].

### 2.4. Measures

Program delivery measures included annual completion rates (%) for each family-facing component: phone calls, initial condolence cards, 3–4-month resource packets, first-anniversary cards, holiday cards, and bereavement books. Completion was defined as documented sending to an eligible family, with eligibility based on the year of death. The program does not track returned packets or undelivered cards; completion reflects documented sending rather than confirmed receipt by the family. The goal was to reach 100% completion per component for eligible families. Some components for 2025–2026 deaths have not yet reached their intended delivery window at the time of data extraction.

Timeliness of outreach was measured as the number of days from the child’s date of death to each bereavement event among eligible families who received that component.

Demographic characteristics included sex, race, ethnicity, preferred language, and primary insurance type. Clinical characteristics included diagnosis category, age at diagnosis, and age at death. End-of-life measures—hospice enrollment, do-not-resuscitate (DNR), Physician Orders for Life-Sustaining-Treatment (POLST) orders, and hospital days in the last 90 days of life—were collected as contextual descriptors of the population served.

### 2.5. Data Collection and Analysis

Bereavement outreach data for all pediatric oncology patients followed by the PPO program were entered into the REDCap database in real time by the clinician (phone call) or PPO practice operations coordinator (mailed items). Data for the current analysis was extracted on 22 May 2026. Demographic, clinical, end-of-life, and bereavement outreach variables were obtained from the same database.

Descriptive statistics were used to characterize the study population and program delivery. Categorical variables were summarized as frequencies and proportions. Continuous variables were summarized as medians with interquartile ranges (IQR), as appropriate. Annual completion rates were calculated for each component by year of death among eligible families, and timeliness was reported as the median number of days from death to each bereavement event.

Exploratory analyses were planned to evaluate completion rates by diagnosis, race/ethnicity, insurance type, and program era (pre- vs. post-standardization). Statistical analyses were performed using RStudio version 4.4.0.

This project was conducted as a quality improvement initiative using de-identified data from deceased patients. Institutional review status was determined to be non-human subjects research, as all patients included are deceased.

## 3. Results

### 3.1. Cohort Characteristics

A total of 403 deceased patients with cancer followed by the PPO program between 2017 and 2026 were included. The cohort was diverse: 41% Black, 15% Hispanic/Latino, and 50% Medicaid-insured, with a median age at death of 13.5 years (IQR 7.4–18.2) (Table 1). Eighty percent had a DNR or POLST order, and 70% were enrolled in hospice. Of the 299 patients with a documented preferred location of death, 87% achieved concordance between preferred and actual location of death (Table 1).

### 3.2. Program Evolution and Bereavement Outreach Delivery

Bereavement outreach delivery evolved substantially over the study period, reflecting sequential implementation of program components through an iterative QI framework (Figure 1).

During the pre-implementation period from 2017 to 2019, bereavement phone calls were the only systematically tracked outreach component. Annual phone call completion rates during this period were 75.0% in 2017, 74.2% in 2018, and 81.1% in 2019. No other family-facing bereavement activities were consistently documented during this phase (Table 2, Supplementary File S1).

Following program standardization in 2020, bereavement outreach expanded from a single tracked activity to a multi-component program, with phone call completion increasing from 74–81% to 94–100% annually (Table 2). Initial condolence cards achieved 98–100% completion through 2026, and first-anniversary cards reached 86–98%, with the lowest rate occurring in 2021 during a period of staff turnover and REDCap documentation inconsistency.

**Figure 2:**
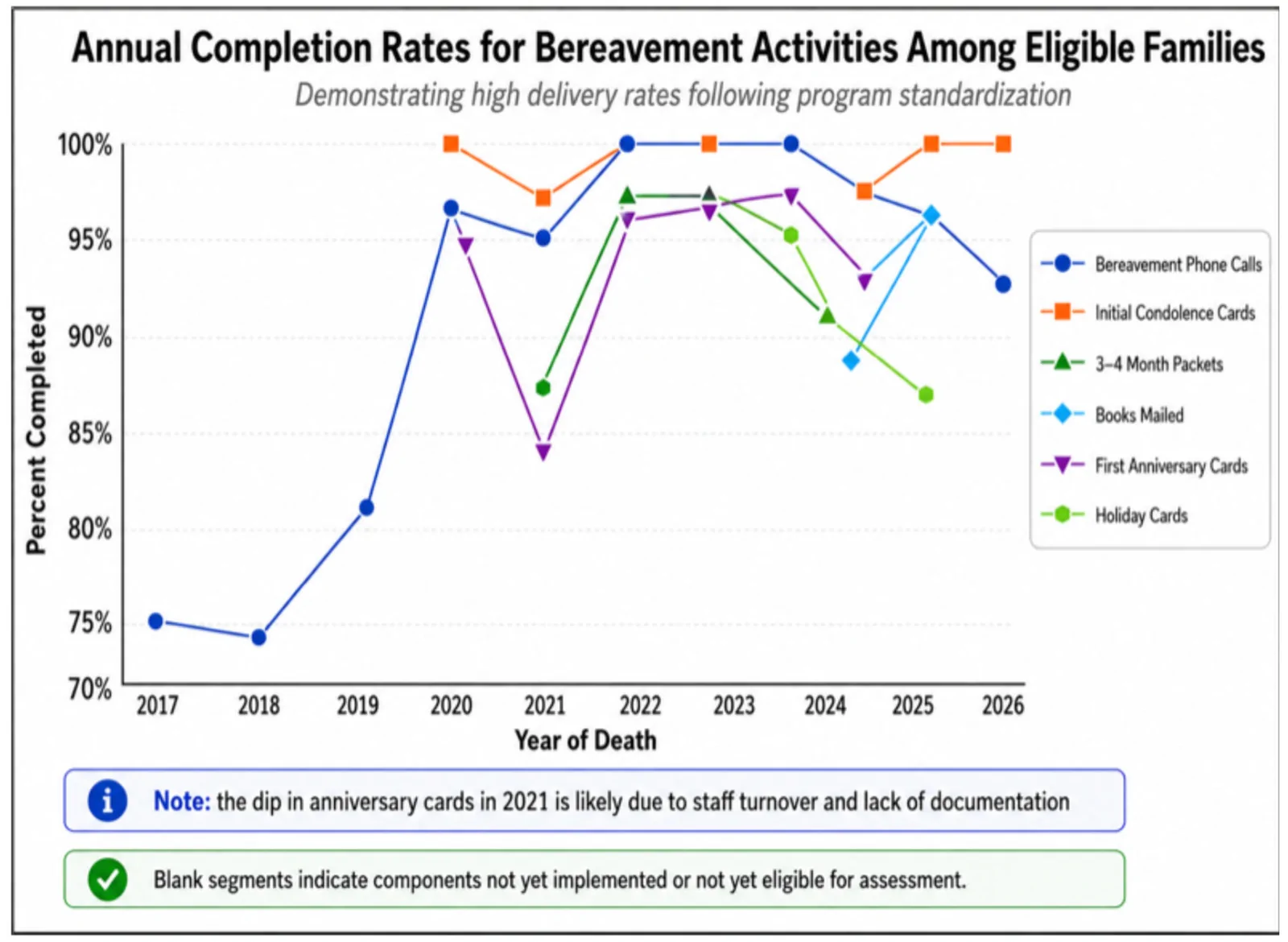
Annual completion rates for bereavement program components by year of death (2017–2026). Each line begins at the year the component was implemented. Lines may visually overlap when completion rates are similar or identical across components in a given year; this does not indicate missing data. Exact values for all components are reported in Table 2. Data from 2025 and 2026 should be interpreted in the context of incomplete follow-up windows at the time of data extraction (22 May 2026).

Additional longitudinal components showed similarly high uptake: the 3–4-month resource packet sustained 93–98% completion after 2021, and holiday cards maintained 88–100% completion after 2022. Bereavement books, added in 2023, rose from 16.0% to 97% (2025) after dedicated procurement was established. Annual completion rates for all components are presented in Table 2 and Figure 2.

### 3.3. Timeliness of Bereavement Outreach

Among eligible families, early outreach occurred promptly: phone calls and condolence cards were both completed at a median of 6 days after death (IQR 4–10). Longer-term outreach also occurred within the intended windows: resource packets at a median of 96 days and first-anniversary cards at 358 days after death (Table 3).

### 3.4. Effect on System-Wide Bereavement Efforts

In 2022, the inpatient PPC team added a dedicated bereavement coordinator to support families served by the inpatient service—a role distinct from the outpatient PPO program described here. This coordinator assists with calls, cards, annual memorial services, and family programming for inpatient PPC families. The development of the outpatient PPO bereavement program informed and provided a model for the creation of this inpatient coordinator role. The hospital system also subsequently developed a Bereavement Care Program team in 2024 including 1.75 staff members; their program includes a Provider Advisory Council (2025-), a Family Advisory Council (2025-) which includes two PPO bereaved parents, and condolence cards and initial bereavement phone calls (2026-).

## 4. Discussion

### 4.1. Key Findings

This retrospective analysis demonstrates that a structured, iteratively developed bereavement program can achieve high and sustained multi-component delivery across a diverse pediatric oncology cohort. These findings compare favorably with published national survey data demonstrating persistent gaps and inconsistent delivery of bereavement cards, phone calls, and services across pediatric oncology programs [16,17]. In contrast, the CHOA PPO program is achieving consistent delivery rates of 94–100%.

### 4.2. Mapping Program Components to National Standards

Multiple professional organizations endorse bereavement care as a standard component of pediatric oncology, palliative care, and end-of-life care. However, guideline recommendations are broad and may be difficult for programs to translate into practice. Table 4 maps the components of the PPO bereavement program to recommendations from the AAP, NCCN, Psychosocial Standards for Children with Cancer and Their Families, and Society of Critical Care Medicine guidelines.

The program operationalizes these national recommendations through concrete, measurable activities. Family-facing components include a bereavement phone call within two weeks of death, an initial condolence card, a 3–4-month bereavement resource packet, a first-anniversary card, a holiday card, and age-appropriate bereavement books for parents and siblings. Parents who are over six months out from their child’s death are offered participation in support groups; this threshold allows for passage through the acute grief period and alignment with established bereavement support timelines [14]. Together, these components provide multiple structured touchpoints across the first year after a child’s death, exceeding the NCCN AYA Oncology minimum of at least one post-death contact and extending support beyond the immediate period of loss [14]. Importantly, as 70% of PPO patients also receive home hospice services, their families receive an additional 13+ months of bereavement support provided by the hospice agency. Staff-facing components, including a monthly neuro-oncology bereavement pause, annual team bereavement event, and involvement in the annual Celebration of Life and Bereavement Provider Advisory Council, further align with recommendations for clinician reflection, staff support, and memorial practices.

Mapping our program components to national guidelines highlights an important implementation principle: national standards become more actionable when translated into specific workflows with defined timing, responsible team members, documentation processes, and measurable delivery outcomes. Multiple team members are clear about their individual responsibilities, and the program uses a combination of Microsoft Teams and REDCap to track outcomes. By linking each program component to guideline recommendations, Table 4 provides a framework that other pediatric oncology and palliative care programs may adapt when building or strengthening bereavement services.

### 4.3. Comparison to Published Bereavement Programs

These findings extend prior descriptions of pediatric bereavement programs. The St. Jude bereavement program established a comprehensive coordinator-driven model with parent-created materials and family engagement in research [19], including a bereaved parent mentoring program. The present program does not aim to match this breadth, but contributes longitudinal delivery metrics across 403 families over nine years, demonstrating that a multi-component program can be delivered reliably at scale.

In an Australian bereavement program, parents rated services as highly impactful, yet many were unaware of available components, underscoring the need for systematic communication and delivery [20]. Helton et al. similarly found that bereaved parents value individualized support rather than a “one-size-fits-all” approach [21,22]. The present program addresses these needs through a standardized 3–4-month bereavement resource packet, as well as unique age-specific grief materials, personalized condolence letters, and an annual Celebration of Life.

The evidence on condolence letters as a stand-alone intervention is mixed. A multicenter study found a condolence letter offering post-mortem consultation reduced grief symptoms among adult oncology families, but an adult intensive care unit (ICU)-based trial found a letter alone did not reduce, and may have worsened, grief symptoms [26]. This difference may reflect the lack of a prolonged clinician-family relationship in the ICU setting, in contrast to the longitudinal relationship that develops over the course of pediatric oncology care. Timing and the depth of the relationship likely matter as much as the letter itself; in the PPO program, personalized condolence cards were one component of a longitudinal program rather than an isolated intervention. Families in the present cohort had a median (IQR) of five (2, 9) clinic visits and two (1, 4) phone contacts with the PPO team before death, reflecting a depth of relationship likely different from the single-encounter or short-stay relationships typical of the ICU setting.

### 4.4. System-Level Impact

Beyond its direct delivery to families, the outpatient PPO program informed broader institutional practice: the inpatient PPC service hired a dedicated bereavement coordinator and adopted an expanded condolence card practice modeled on the PPO approach, first adding holiday cards and a service for families, and the institution later initiated a Bereavement Care Program with separate Provider and Family Advisory Councils, and its own system-wide condolence card process. This illustrates how a well-documented program within one area of the hospital can support broader culture change. For institutions building bereavement infrastructure with limited resources, starting with a single motivated team within oncology, cardiology, emergency, or the intensive care units and expanding outward may be more practical than institution-wide implementation from the outset.

### 4.5. Equity Considerations

Equity issues persist into bereavement. The patient population we served was diverse with many Black, Hispanic/Latino, and Spanish-speaking families that were Medicaid-insured [15]. Evidence suggests that Black and Hispanic/Latino bereaved parents whose child died in intensive care settings are at greater risk for poorer adjustment outcomes in bereavement, a finding that may be explained in part by lower therapeutic alliance with medical staff compared to White parents or being put in a more active decision-making role than desired [27,28,29]. With PPC and bereavement literature disproportionately centered on white families, much of the existing evidence on bereaved parents may not be generalizable to the families our program serves [30].

Several program features may help reduce barriers to access: bereavement phone calls do not require families to initiate contact, and the resource packet is mailed directly to the home. For Spanish-speaking families, all written material, including condolence cards, the personalized letter, the When You Are Grieving booklet, and Mourner’s Bill-of-Rights wallet cards, are provided in Spanish. Phone calls occur with an interpreter or are completed by a bilingual clinician. Our program does not track returned packets or cards. As completion rates were consistently high, we could not evaluate whether completion rates differed by race, ethnicity, language, or insurance status. Future work should explicitly examine equity in bereavement delivery across these dimensions, and research conducted with marginalized populations remains essential to understanding how social disparities shape the long-term psychosocial experience of bereavement [30].

### 4.6. Staff Well-Being

Bereavement care and team-based programming also support clinician well-being by providing closure and strengthening team cohesion [2]. Pediatric oncology and palliative care clinicians are at risk for moral distress and compassion fatigue, and the NCCN Guidelines recommend staff debriefing after a patient’s death [14]. The monthly neuro-oncology bereavement pause and the annual PPO team bereavement event address this need, though clinician outcomes are not formally measured.

### 4.7. Challenges and Lessons Learned

Several practical lessons emerged during program development. First, eliciting formal feedback from bereaved families remains challenging. Although families have anecdotally expressed appreciation for condolence cards, first-anniversary cards, and continued communication with the team, systematic data on family perceptions are not yet available. Second, the trajectory of bereavement book delivery illustrates the operational gap between program design and reliable implementation: consistent delivery was not possible until dedicated funds and procurement were established in 2024. Adding a new component requires dedicated personnel and logistical infrastructure, not just clinical intent. Third, the COVID-19 pandemic required adaptation of communal activities: the annual Celebration of Life moved to a virtual format in 2020–2021. The program’s ability to sustain outreach through this disruption suggests standardized workflows help preserve continuity during operational disruption.

### 4.8. Limitations

This quality improvement project has several limitations. First, it is a single-center, retrospective analysis, and findings may not be generalizable to institutions with different staffing or resources. Second, the project reports delivery metrics rather than receipt or impact: documentation confirms an outreach component was completed but not whether the family received that component or found it acceptable and helpful. Third, no formal family-reported outcomes, such as validated grief measures or satisfaction surveys, were collected, limiting our ability to determine whether the program improved psychosocial well-being. The project also did not collect informal or formal feedback from parents regarding their perceptions of, or satisfaction with, these bereavement efforts, which represents an important gap for future work. This will be part of support group programming, providing new parent-reported data. During 2017–2019, only phone calls were systematically tracked, so other informal outreach may be underestimated. The cohort included only patients followed by the PPO program and represents most, though not all, pediatric oncology deaths at the institution. Notably, a related quality improvement effort at our institution demonstrated substantial gains in palliative care access among all deceased pediatric oncology patients over time, with receipt of PPC increasing from 31% to 78% following the creation of an outpatient PPO clinic [31]. When outpatient PPO was unavailable, 69% of patients died without receiving palliative care; this dropped to 22% following the availability of the clinic (*p* < 0.001). Finally, 2025–2026 data should be interpreted cautiously, as some components had not yet reached their intended delivery window at extraction. Missing data were minimal overall; the primary source of incomplete data was the expected follow-up window limitation for 2025–2026 deaths rather than documentation gaps.

### 4.9. Future Directions

Bereaved family feedback on individual component perceived impact, timing, and unmet needs will be needed and could be conducted via survey or qualitative interviews. This would elucidate which families desire more intensive support such as counseling or peer connection. Future work should explore different types of support groups and adaptation of the program to non-oncology palliative care populations. Multi-center collaboration could facilitate shared benchmarks and cross-institutional quality improvement efforts in bereavement care [32]. Finally, future analyses should evaluate equity in bereavement delivery by stratifying completion rates and timeliness by race, ethnicity, preferred language, and insurance status. Prospective studies incorporating validated psychosocial outcome measures, such as the Prolonged Grief-Disorder-13-Revised (PG-13-R), Inventory of Social Support and measures of anxiety and depression (i.e., GAD-7; PHQ-8), could further assess whether structured bereavement programs are associated with improved psychosocial outcomes among bereaved parents and siblings [33].

## 5. Conclusions

This quality improvement project describes the iterative development of a structured, multi-component bereavement program within a large, diverse outpatient pediatric palliative oncology population. Over nine years, the program evolved from informal phone calls to a six-component program reaching excellent annual delivery rates [16,17]. The trajectory of this program demonstrates that comprehensive bereavement care does not require a fully developed infrastructure at the outset. Beginning with a single feasible, trackable activity and establishing reliable documentation allows for sequential addition of components as staffing, workflows, and institutional support evolve. This bereavement program provides a practical and replicable roadmap for programs at any stage of development and illustrates how system-level change can be informed.

## Figures and Tables

**Table 1:** Cohort demographics and clinical characteristics (N = 403).

Characteristic	N = 403
**Sex**	
Female	184 (46%)
Male	216 (54%)
Non-binary	3 (0.7%)
**Race**	
White	204 (51%)
Black	165 (41%)
Asian	22 (5.5%)
Other	12 (3.0%)
**Ethnicity**	
Latino/Spanish/Hispanic	61 (15%)
Non-Latino	342 (85%)
**Age at diagnosis (years)**	
Median (Q1, Q3)	9.87 (4.48, 14.82)
**Primary insurance type**	
Medicaid	201 (50%)
Private	170 (42%)
Tricare	12 (3.0%)
Other/Uninsured	20 (5.0%)
**Preferred language**	
English	360 (89%)
Spanish	38 (9.4%)
Other	5 (1.2%)
**Diagnosis type**	
Solid tumor	170 (42%)
Brain tumor	158 (39%)
Leukemia/lymphoma	65 (16%)
Hematologic	6 (1.5%)
Other	4 (1.0%)
**Age at death (years)**	
Median (Q1, Q3)	13.45 (7.44, 18.17)
**End-of-life characteristics**	
Any EOL order (DNR or POLST)	321 (80%)
Enrolled in hospice	283 (70%)

**Table 2:** Bereavement outreach delivery rates by component and year of death.

Year of Death	Number of Deaths	Bereavement Phone Calls, n (%)	Initial Condolence Cards, n (%)	3–4 Month Packets, n (%)	Books Sent, n (%)	Anniversary Cards, n (%)	Holiday Cards, n (%)
2017	8	6 (75.0%)	—	—	—	—	—
2018	31	23 (74.2%)	—	—	—	—	—
2019	37	30 (81.1%)	—	—	—	—	—
2020	35	34 (97.1%)	35 (100.0%)	—	—	34 (97.1%)	—
2021	51	49 (96.1%)	50 (98.0%)	45 (88.2%)	—	44 (86.3%) ^b^	16 (31.4%) ^c^
2022	47	47 (100.0%)	47 (100.0%)	46 (97.9%)	—	46 (97.9%)	47 (100.0%)
2023	50	50 (100.0%)	49 (98.0%)	49 (98.0%)	8 (16.0%) ^d^	49 (98.0%)	50 (100.0%)
2024	60	59 (98.3%)	59 (98.3%)	56 (93.3%)	54 (90.0%)	56 (93.3%)	56 (93.3%)
2025 ^a^	67	65 (97.0%)	67 (100.0%)	65 (97.0%)	65 (97.0%)	17 (25.4%) ^a^	59 (88.1%)
2026 ^a^	17	16 (94.1%)	17 (100.0%)	1 (5.9%) ^a^	1 (5.9%) ^a^	0 (0.0%) ^a^	0 (0.0%) ^a^

“—” indicates the component had not yet been implemented and families were not eligible. ^a^ The lower number of deaths in 2017 reflects a partial year of data; the outpatient PPO program launched in July 2017. The year 2018 represents the first full year of program operation. Data from 2025 and 2026 should be interpreted in the context of incomplete follow-up windows at the time of data extraction (22 May 2026). Anniversary cards for 2025 deaths and most components for 2026 deaths had not yet reached their intended delivery window. ^b^ The lower anniversary card rate in 2021 is likely attributable to documentation inconsistency rather than failure to send. ^c^ Holiday cards were not formally implemented until 2022; the 2021 figure reflects partial early adoption. ^d^ Books were formally added in 2023 but were initially limited by supply; consistent delivery was achieved after dedicated procurement in 2024.

**Table 3:** Timeliness of bereavement outreach activities (eligible patients only).

Bereavement Activity	Median Days from Death (IQR)
Bereavement phone call	6 (4, 10)
Initial condolence card	6 (4, 10)
3–4 month bereavement packet	96 (84, 108)
First-anniversary card	358 (350, 365)

**Table 4:** Mapping bereavement program components to national guideline recommendations.

Program Component	Timing	AAP (Linebarger 2022; Weaver 2023) [11,12]	NCCN [14]	Psychosocial Standards (Lichtenthal 2015) [2]	SCCM (Derrington 2026) [15]
Bereavement phone call	Within 2 weeks of death	Phone call to family; follow-up to assess coping [11,12]	Formally express condolences (card, call, letter); assess for complicated grief [14]	Contact bereaved families to assess needs and identify at-risk individuals [2]	Bereavement care process during or after death [15]
Initial condolence card	At time of death	Condolence letter; acknowledge the death [11,12]	Formally express condolences (card, call, letter) [14]	Contact bereaved families [2]	Bereavement care process after death [15]
3–4 month bereavement resource packet	3–4 months after death	Inform families about resources; sibling support; referral to counseling [11,12]	Refer to bereavement services; assess for complicated grief [14]	Provide bereavement resources; identify at-risk families [2]	Emotional, informational, and practical assistance; tailored to family needs [15]
First-anniversary card	~12 months after death	Grief has no finite timeline; ongoing support [11,12]	Periodically assess and monitor for complicated grief [14]	Continued care over time [2]	—
Holiday card	First holiday season after death	Memorial services and remembrances [11]	Memorial ritual for staff and families [14]	—	Memory-making activities [15]
Age-specific books	Included in resource packet	Sibling support; age-appropriate grief guidance [11,12]	—	Sibling-specific bereavement support [2]	Services tailored to child age and developmental level [15]
Neuro-oncology bereavement pause (staff)	Monthly	Clinician debriefing [11]	Staff debriefing; identify clinicians at risk for compassion fatigue; bereavement ritual for staff [14]	—	—
Celebration of Life	Invited year after death	Memorial services; Sibling support [11]	Memorial ritual; bereavement ritual for staff [14]	—	Memorial services; Memory-making activities [15]

AAP = American Academy of Pediatrics; NCCN = National Comprehensive Cancer Network; SCCM = Society of Critical Care Medicine; “—” indicates the guideline does not include a specific recommendation corresponding to this component.

## Data Availability

The data supporting the findings of this study are not publicly available due to patient privacy considerations but deidentified data may be available upon reasonable request and with appropriate institutional approvals.

## Supplementary Materials

The following supporting information can be downloaded at https://www.mdpi.com/article/10.3390/children13091163/s1, Figure S1: Teams Clinician Shared Task List for Bereavement Tracking. Figure S2: REDCap Bereavement Component Tracking. Table S1: Contents of the 3–4 month bereavement resource packet. File S1: Bereavement Phone Call Guidance [34,35,36,37].
