# Supplementary material for: Implementation and Delivery of a Comprehensive Bereavement Program in Pediatric Palliative Oncology
Source: Children (Basel). 2026 Aug 29;13(9):1163. doi: 10.3390/children13091163 (PMC13605475; doi:10.3390/children13091163)
Supplement: Supplementary file 1 [file children-13-01163-s001.zip › children-4469549-supplementary.pdf]

Supplement

Figure S1. Teams Clinician Shared Task List for Bereavement Tracking

Bereavement ☆ All Items

Patient	Call Assigned to	Cards Sent	Call Done/ Documented	Redcap closed	EPIC/ mark dcsd	Emails (Onc/PACT)	Notes
Patient A.B.	Lange, Anna			✓	✓	✓	
Patient C.D.	Brock, Katharine	✓	✓	✓	✓	✓	

Figure S2. REDCap Bereavement Component Tracking

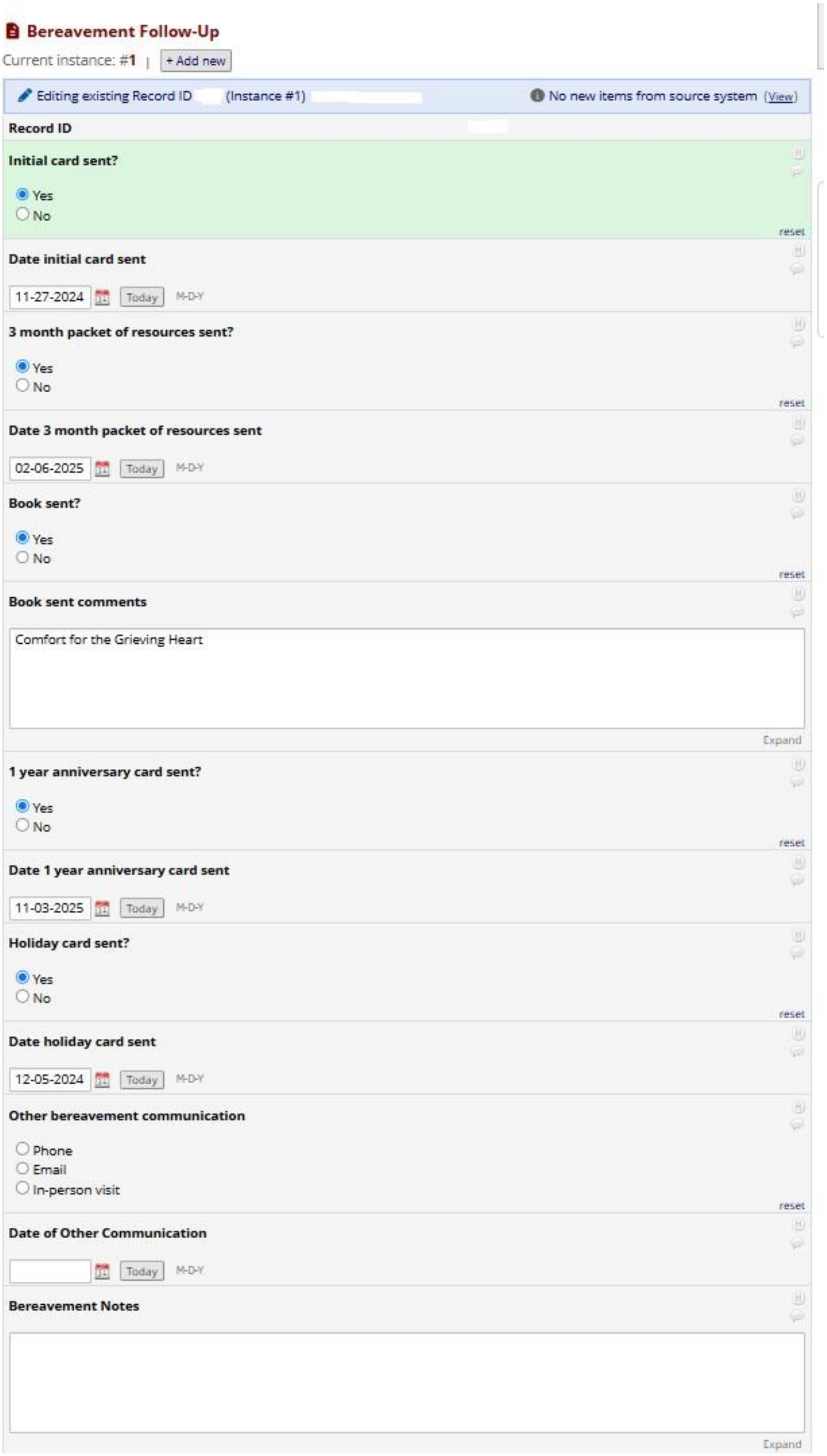

Bereavement Follow-Up

Current instance: #**1** | + Add new

Editing existing Record ID (Instance #1) — No new items from source system (View)

Record ID

Initial card sent?
- (•) Yes
- ( ) No

reset

Date initial card sent

11-27-2024 Today M-D-Y

3 month packet of resources sent?
- (•) Yes
- ( ) No

reset

Date 3 month packet of resources sent

02-06-2025 Today M-D-Y

Book sent?
- (•) Yes
- ( ) No

reset

Book sent comments

Comfort for the Grieving Heart

Expand

1 year anniversary card sent?
- (•) Yes
- ( ) No

reset

Date 1 year anniversary card sent

11-03-2025 Today M-D-Y

Holiday card sent?
- (•) Yes
- ( ) No

reset

Date holiday card sent

12-05-2024 Today M-D-Y

Other bereavement communication
- ( ) Phone
- ( ) Email
- ( ) In-person visit

reset

Date of Other Communication

Today M-D-Y

Bereavement Notes

Expand

Table S1. 3-4 month post-death resource packet contents:

- Personalized condolence letter signed by the team (see below)
- Community resource list (see below)
- Age-specific sibling grief information (see below example)
- *When You Are Grieving* booklet
    - *https://www.qolpublishing.com/booklets/1217-2/*
- Mourner's Bill of Rights
    - *https://www.centerforloss.com/bookstore/mourner-s-bill-rights-wallet-cards/*
- Books (all stamped with "Supportive Care Clinic"):
    - Parent Options
        - Shattered: Surviving the Loss of a Child by Gary Roe
        - Comfort for the Grieving Parent's Heart: Hope and Healing After Losing Your Child by Gary Roe
        - Cómo Superar la muerte de alguien que amas (Spanish) by Diana Baker
    - Teen Options:
        - It Won't Ever be the Same by Korie Leigh
        - The Grief Thing: A Guided Grief Journal for Teens Navigating Loss by Rebecca Black
        - Fire in My Heart, Ice in My Veins by Enid Samuel Traisman
        - How I Feel: Grief Journal for Kids by Mia Roldan
        - Healing Your Grieving Heart for Teens by Alan Wolfelt
    - Younger Children Options:
        - Gift of Gerbert Feathers by Lori Weiner and Meaghann Weaver
        - Ida Always by Caron Levis
        - When Someone Very Special Dies by Marge Heegaard
        - Lifetimes by Bryan Mellonie
        - I Miss You by Pat Thomas
        - The Invisible String (English and Spanish) by Patrice Karst
        - The Memory Box by Joanna Rowland

Dear family of ***,

Words cannot express how sorry we are for your loss. *** was so brave and joyful. We miss *** every day, and our thoughts are with you and your family.

You may feel lost and lonely after the loss of your child. Our hearts are with you. You may also grieve the loss of *** (his/her) healthcare team and your role as a caregiver. You are not alone. We are here for you and want to connect you with resources that you may find helpful in your grief journey.

In this packet, we have included some resources that other parents have found helpful:

- When You Are Grieving: A Guide to Understanding Loss
- The Mourner's Bill of Rights. This is a reminder that:
  - Each person's grief is unique.
  - You have the right to move toward your grief and healing.
- Local resources list. This includes more details about:
  - Counseling, Camps & Books for children and adults
  - Online resource guides & support videos
- Sibling grief
  - How to support your child's grief based on their developmental stage.

Grief-related thoughts, behaviors and feelings can be very distressing and relentless. You may experience stress after your loss, with symptoms such as: crying at unexpected times, guilt, anger and feeling as if it is not real. Some ways to help cope with stress include joining support groups, sharing your thoughts, and seeking therapy.

Symptoms of grief can become life threatening, especially if they do not improve after many months. If you feel overwhelmed and have thoughts of hurting yourself or someone else, please:

- Call 911 right away. Ask for a CIT (crisis intervention trained officer).
- Call the Georgia Crisis and Access Line at 1-800-715-4225 or visit www.mygcal.com.
- Call the Suicide & Crisis Lifeline at 988 for free and confidential emotional support to people in suicidal crisis or emotional distress 24 hours a day, 7 days a week, across the United States and its territories.

Please call 404-785-1200 to reach your primary social worker at Children's if you are in distress or need more resources to help cope with your loss.

*** will always be remembered by our team.

With love and support,

Supportive Care Clinic
Katharine Brock, MD, MS, Director
Jonathan Ebelhar, MD, MSc
Anna Lange, MSN, RN, CPNP
Karyn Korsah, MD – Psychiatry
Linda Radbill, PhD – Psychology

Resources to Support You

This handout lists support programs that may help meet the needs of families. When you contact a program, make sure to ask questions about the program's philosophy, group structure, fees and goals. Please do not hesitate to reach out to your primary social worker or email aflacsupportivecare@choa.org if you need further help.

- Kate's Club (Atlanta)
  - katesclub.org
  - Kate's Club has services for children, teens and adults.
  - Services for children and teens include:
    - Monthly outings in the Atlanta area
    - Age-based support groups
    - Art and music activities
    - Camp Good Mourning (an annual bereavement camp)
- Camp Sunshine (Atlanta area)
  - mycampsunshine.org
  - Remember the Sunshine Family Night happens the first Thursday evening of each month.
- Chase the Victory (Southeast)
  - Parent support groups both in person (Carrollton, Georgia) and virtually
  - Family & Marriage Retreats for Bereaved Families – located both in Panama City Beach, FL & Atlanta
  - 2026 Dates Available
  - Chase The Victory – Share Hope. Spread Kindness.
- Cure Childhood Cancer – Family Resources – Counseling & Bereavement
  - Grief Counseling - CURE provides sessions for the parent or sibling of eligible families. The first session is free; for each session thereafter, the family will pay the therapist directly a $25.00 co-pay per session. Sessions may be used in any combination to best support the family's needs: individual counseling, couples counseling, family counseling, etc. You can apply for counseling on their website or reach out to Chelsea Key (770-986-0035 ext. 39 or chelsea@curechildhoodcancer.org) directly to get connected with a therapist in your community.
  - Weekend of Hope and Healing – time geared toward families who have lost a child to pediatric cancer in the last three years. Together we celebrate the lives of children who are no longer with us while providing support, comfort, guest speakers, and breakout sessions to assist parents in their grieving process.
  - Quarterly fellowship evenings for fathers who have lost a child to cancer.
  - For information about our bereavement support, please email Karen Rutherford at karen@curechildhoodcancer.org.
  - www.curechildhoodcancer.org
- The Link Counseling Center (Atlanta, Sandy Springs and Cobb county)
  - thelink.org
  - This center has weekly support groups for children and teens that have lost a loved one.
  - It has quality and affordable programs for all ages. Programs include confidential counseling, psychotherapy and support groups.

- Hope for Grieving Children (Roswell)
  - hopeforgrievingchildrengeorgia.org
  - Hope for Grieving Children offers six-session support groups for both children and parents.
- ThriveWorks (Alpharetta, Athens, Atlanta [Buckhead, Duluth, Smyrna], Cumming, Dallas, Decatur, Kennesaw, Lawrenceville, Marietta, McDonough, Peachtree City, Pooler, & Woodstock)
  - Grief counseling and psychiatry – offers individual & couples
  - In network with most insurance plans
  - https://thriveworks.com/
- Compassionate Friends (all over Georgia)
  - Compassionate Friends helps grieving families after the death of a child of any age.
  - Support groups meet throughout Georgia for both parents and siblings (ages 12 and up).

Camps

- Camp Erin / Camp MAGIK (Atlanta, Hampton, Cartersville and Union Point)
  - campmagik.org
  - Camp Erin is a bereavement camp for children and teens that have lost a loved one.
  - It is for children ages 6 to 17. Free of charge.
  - It lets children and teens express their grief and start the healing process.
- Camp Good Mourning (Atlanta)
  - Katesclub.org
- Camp Stars (Atlanta area)
  - vnhs.org/hospice-atlanta
  - Camps Stars is a weekend bereavement camp for children and families.
  - It is sponsored by Hospice Atlanta.
- Camp Sunshine (Atlanta area)
  - mycampsunshine.org
- Camp Hope (Jesup)
  - Hospiceoga.org
- Experience Camps (Northeast Georgia)
  - experience.camp
  - This is a one-week camp for boys and girls who have lost a parent, sibling or primary caregiver.

Books and literature

Storybooks

- The Invisible String by Patrice Karst (ages 3 and up)
  - This book offers a gentle approach to help overcome the fear of loneliness or separation.
  - It is a creative book that children can easily identify with and remember.
- The Memory Box by Joanna Rowland
  - A Book about Grief
  - Describes what it is like to remember and grieve a loved one who has died.
- The Next Place by Warren Hanson (ages 4 and up)
  - This is a story of the next place, a place of light and hope where earthly hurt is left behind. It is a celebration of life.
  - This story presents death in a non-secular (religious) way.
- When Dinosaurs Die: A Guide to Understanding Death by Laurie Kransy Brown and Marc Brown (ages 4 to 10)

  - This is a guide to help children understand what death means and how to cope with feelings.
- Lifetimes by Bryan Mellonie (ages 5 to 10)
  - This is a story about life and death.
  - It uses nature, animals and people.
  - It talks about beginnings, endings and living in between.
- The Fall of Freddie the Leaf by Leo Buscaglia (ages 5 to 9)
  - This is a story about a leaf named Freddie.
  - It describes the seasons of life and death through nature.
- Sad Isn't Bad by Michaelene Mundy (ages 3 to 10)
  - A good-grief guidebook for kids dealing with loss.
- The Gifts of Gerbert's Feathers by Meaghann Weaver & Lori Weaver (ages 4 to 8)
  - A delightful story about strength, courage, determination, and love. Used to help children dealing with an illness or those who are worried about others with a serious illness.

Books for Teens

- Healing Your Grieving Heart for Teens by Alan Wolfelt (ages 12 to 18)
  - 100 Practical Ideas that can help survivors learn to express their grief and mourn naturally.
  - A journal for teenagers experiencing loss. Teens can write letters, copy down meaningful lyrics, write songs and poems, tell the person who died what they want them to know, and use their creativity to work through the grieving process.
- Fire in My Heart, Ice in My Veins by Enid Samuel Traisman (ages 12 to 18)
  - 100 Practical Ideas that can help survivors learn to express their grief and mourn naturally.

Activity books

- How I Feel: A Coloring Book for Grieving Children by Alan D. Wolfelt, Ph.D. (ages 3 to 8)
  - This is a coloring book that shows the many feelings grieving children may have.
  - You can use this book as a guide to help your child share his feelings.
- When Someone Very Special Dies by Marge Heegaard (ages 3 to 10)
  - A practical format for allowing children to understand the concept of death and develop coping skills for life.
  - Children can learn to cope with grief (drawing out feelings)
- Help Me Say Goodbye: Activities for Helping Kids Cope When a Special Person Dies by Janis Silverman (Ages 4 to 12)
  - This is an art therapy book. It encourages children to express their feelings in words or pictures.
  - This book goes over many topics, such as: what to say or do, feelings, how to remember your loved one
- How I Feel: Grief Journal for Kids by Mia Roldan (ages 8 to 12)
  - Guided prompts to explore your feelings and find peace
  - Give the child in your life the emotional tools to start healing from their loss
- When Someone has a Very Serious Illness by Marge Heegaard (ages 9 to 12)
  - An excellent resource for helping children learn the basic concepts of illness and various age-appropriate ways of coping with it.

Care Packages

- Eluna Cares (ages 6 to 17)

- Eluna Cares offers personalized care packages to children and teens ages 6-17 impacted by grief. Eluna Cares is a tangible reminder that we can see those grieving – we hear them – and we are here to support and guide them through difficult life events.

Parent resources

- Momento Foundation
  - Non-profit dedicated to creating free grief resources and community tools. Resource list includes – a bookshelf, podcasts, and articles + blogs.
- www.momentofoundation.org

Parent Books

- Bearing the Unbearable by Joanne Cacciatore
- Comfort for the Grieving Parent's Heart: Hope and Healing After Losing Your Child by Gary Roe
- Shattered: Surviving the Loss of a Child by Gary Roe
- Shelter from the Storm: Caring for a Child with a Life Threatening Condition by Joanne Hiblen, Karen Lindsey and Daniel Tobin
- When the Bough Breaks: Forever After the Death of a Son or Daughter by Judith Bernstein, PhD
- A Different Dream for my Child by Jolene Philo
- Safe Passage: Words to Help the Grieving Hold Fast and Let Go by Molly Fumia
  - This is a book of meditations on grieving.
- The Worst Loss: How Families Heal from the Death of a Child by Barbara D. Rosof
  - This is a book on parental bereavement.

Grief books for men

- The Way Men Heal by Thomas Golden
- Swallowed by a Snake: The Gift of the Masculine Side of Healing by Thomas Golden
- Pigtails & Steel by Andy McCall

Online resource guides

- Courageous Parents Network
  - courageousparentsnetwork.org/guided-pathways/supporting-families-in-bereavement/
  - This includes more details on how grief is an ongoing process.
- Good Grief
  - good-grief.org/
- National Alliance for Grieving Children
  - childrengrieve.org/resources/about-childhood-grief
  - This is about childhood grief.
- Understanding Death, Grief, & Mourning: A Resource Manual
  - my.clevelandclinic.org/ccf/media/Files/bereavement/understanding-death-grief-mourning-resources-manual.pdf?la=en
  - This manual includes many topics, such as:
    - Talking to children
    - Children and bereavement
    - How grief may look in children
    - Helping children cope

    - How to tell if your child needs counseling
    - Books for children and teens
    - Books for adults
    - Coping as a family
    - Adult grief
    - Grief and the scriptures
- Parenting After Child-Loss
    - https://courageousparentsnetwork.org/guides/parenting-after-child-loss

Support videos

- Courageous Parents Network
  - courageousparentsnetwork.org/video-library/bereavement-and-legacy/bereavement
  - This video includes topics, such as:
    - Finding your child's legacy
    - What to do with your child's things
    - Transformation and meaning
    - Your identity as a parent
    - Caring for Bereaved Siblings

This list was compiled by the Supportive Care Clinic at the Aflac Cancer and Blood Disorder Center

Children's Healthcare of Atlanta has not reviewed all of the sites listed as resources and does not make any representations regarding their content or accuracy. Children's Healthcare of Atlanta does not recommend or endorse any particular products, services or the content or use of any third party websites, or make any determination that such products, services or websites are necessary or appropriate for you or for the use in rendering care to patients. Children's Healthcare of Atlanta is not responsible for the content of any of the above- referenced sites or any sites linked to these Sites. Use of the links provided on this or other sites is at your sole risk.

Grief and death

How to support your preschooler (ages 3 to 5 years)

How might my child respond to grief?

- Your child may try to connect events that are not related. For example:
  - Grandpa may have had a headache and died. Now, your child may fear that Mommy will die because she has a headache.
- Your child may view death as passing. He cannot understand the concept of death. He may think the dead person will come back.
- Your child may:
  - Think that he caused the death by his thoughts, words or actions.
  - Feel insecure.
  - Be scared to be apart from those close to him.
- Your child's language and expressions are limited. Your child may respond to grief with one or more of these:
  - Clinging
  - Whining
  - Hyperactivity (more active than normal)
  - Withdrawal
  - More aggressive than normal
  - Anger

How can I help my child?

There are many things parents or caregivers can do to make death a little easier for your child.

- Use simple words that are true, such as:
  - "John died last night. When someone dies, his body stops working. His body will never work again. This means that John will no longer play or eat dinner with us."
- Tell your child that the death is not his fault and that he did not cause the death to happen by anything he thought or said.
- It is common for grieving children to have behavior changes or behavior that regresses (such as potty training or sleep habits). Most often, these will pass as your child moves forward in the grieving process.
  - Be patient and support your child during these hard times.
  - Keep your daily routine when you can. This can help your child feel more secure.
- Reassure your child with words, such as:
  - "Our family is going to be OK, but we are very sad right now because we miss Jane so much."

This handout contains general information only. Talk with your child's doctor or a member of your child's healthcare team about specific care of your child. In case of an urgent concern or emergency, call 911 or go to the nearest emergency department right away.

Supplementary File S1

Bereavement Phone Call Guidance Document

Outpatient Pediatric Palliative Oncology Program

Aflac Cancer and Blood Disorders Center, Children's Healthcare of Atlanta

TIPS FOR THE CALL

- Unblock your phone number if calling from a CHOA or Emory phone
- Think about the family's routine and determine the best time to call (consider: other children at home, work obligations)
- Use the name of the child when speaking about him/her
- Encourage, but do not pressure, the family into speaking

POTENTIAL INTRODUCTION

- "Hi ____, this is __________ from the Oncology/PACT/Supportive Care team at Children's. I heard that _____ died and I am so sorry for your loss. Our team sends their condolences. We cannot begin to imagine what your family has endured during the past few days/weeks."
- Ask if now is a good time, or if they have a few moments to speak
- Start with a broad question: e.g., "What have your days been like since _____ died?" (avoid sentiments such as "How are you doing?")

POTENTIAL FOLLOW-UP TOPICS

- Ask how they are feeling, both physically and emotionally (eating? sleeping?)
- If there are other family members, ask how they are coping
- Ask if they are feeling supported during this difficult time
- Encourage social connections in relation to building their supports
- If you have a story to share about the child, ask to share it, or ask the family to share a favorite moment with you
- Acknowledge and highlight the family's strengths at this time
- Emphasize that it is okay to experience complex emotions and that grieving looks different for each individual. There is no right way to feel at this time.

ENDING THE CONVERSATION

- Many families do not respond to initial efforts of establishing contact, but are reassured that there is someone they can reach out to in case of a future need. Consider making sure the family has the Oncology or PACT number to call if they want to reach out.
- If true, remind the family that they will be receiving additional resources in the mail and verify their address if possible.

LEAVING A MESSAGE

- If it is an answering machine, consider leaving a message expressing that you are thinking of them and wanted to check in to see how things have been. Leave contact info for Oncology/PACT in the event they wish to reach out.
- It is suggested to make 2 attempts to reach the family.
- Try using a different number the second time (cell vs. home).

REFERENCES

Darbyshire P, Cleghorn A, Downes M, et al. Supporting bereaved parents: a phenomenological study of a telephone intervention programme in a paediatric oncology unit. J Clin Nurs. 2013;22(3-4):540-549.

Hottensen D. Bereavement: Caring for Families and Friends after a Patient Dies. OMEGA-Journal of Death and Dying. 2013;67(1-2):121-126.

Morris SE, Block SD. Adding Value to Palliative Care Services: The Development of an Institutional Bereavement Program. J Palliat Med. 2015;18(11):915-922.

Script for telephone conversation with the bereaved [Pamphlet]. New York, NY: Visiting Nurse Service of New York Hospice Care; 2008.
